# The Impact of *Helicobacter pylori* Urease upon Platelets and Consequent Contributions to Inflammation

**DOI:** 10.3389/fmicb.2017.02447

**Published:** 2017-12-12

**Authors:** Adriele Scopel-Guerra, Deiber Olivera-Severo, Fernanda Staniscuaski, Augusto F. Uberti, Natália Callai-Silva, Natália Jaeger, Bárbara N. Porto, Celia R. Carlini

**Affiliations:** ^1^Center of Biotechnology, Universidade Federal do Rio Grande do Sul, Porto Alegre, Brazil; ^2^Department of Biology, Universidade Regional Integrada do Alto Uruguai e das Missões, São Luiz Gonzaga, Brazil; ^3^Department of Molecular Biology and Biotechnology, Institute of Biosciences, Universidade Federal do Rio Grande do Sul, Porto Alegre, Brazil; ^4^Institute of Biology, Universidade Federal de Pelotas, Pelotas, Brazil; ^5^Institute of Biomedical Research, Pontifícia Universidade Católica do Rio Grande do Sul, Porto Alegre, Brazil; ^6^Brain Institute (BRAINS-InsCer), Pontifícia Universidade Católica do Rio Grande do Sul, Porto Alegre, Brazil

**Keywords:** inflammation, mRNA processing, IL-1β, lipoxygenase inhibitors, CD14, GPVI, collagen receptor, platelet aggregation

## Abstract

Gastric infection by *Helicobacter pylori* is considered a risk factor for gastric and duodenal cancer, and extragastric diseases. Previous data have shown that, in a non-enzymatic way, *H. pylori* urease (HPU) activates neutrophils to produce ROS and also induces platelet aggregation, requiring ADP secretion modulated by the 12-lipoxygenase pathway, a signaling cascade also triggered by the physiological agonist collagen. Here we investigated further the effects on platelets of recombinant versions of the holoenzyme HPU, and of its two subunits (HpUreA and HpUreB). Although HpUreA had no aggregating activity on platelets, it partially inhibited collagen-induced aggregation. HpUreB induced platelet aggregation in the nanomolar range, and also interfered dose-dependently on both collagen- and ADP-induced platelet aggregation. HPU-induced platelet aggregation was inhibited by antibodies against glycoprotein VI (GPVI), the main collagen receptor in platelets. Flow cytometry analysis revealed exposure of P-selectin in HPU-activated platelets. Anti-glycoprotein IIbIIIa (GPIIbIIIa) antibodies increased the binding of FITC-labeled HPU to activated platelets, whereas anti-GPVI did not. Evaluation of post-transcriptional events in HPU-activated platelets revealed modifications in the pre-mRNA processing of pro-inflammatory proteins, with increased levels of mRNAs encoding IL-1β and CD14. We concluded that HPU activates platelets probably through its HpUreB subunit. Activation of platelets by HPU turns these cells into a pro-inflammatory phenotype. Altogether, our data suggest that *H. pylori* urease, besides allowing bacterial survival within the gastric mucosa, may have an important, and so far overlooked, role in gastric inflammation mediated by urease-activated neutrophils and platelets.

## Introduction

Diseases caused by *Helicobacter pylori* have a great impact on public health, since this bacterium colonizes the gastric mucosa of half of the world's population, with a higher prevalence in the poorer countries (Parkin, 2004). *Helicobacter pylori* is a major cause of gastric and duodenal pathologies (Ferlay et al., 2013) and it was classified as the first carcinogenic bacterium by the World Health Organization more than 2 decades ago (IARC, 1994). Urease produced by *H. pylori* enables bacterial colonization of the gastric mucosa by catalyzing the hydrolysis of urea into carbon dioxide and ammonia, thereby causing a local pH increase and alterations of the mucus properties that favor the pathogen's survival (Perrais et al., 2014). Urease-negative strains of *H. pylori* were unable to infect the gastric mucosa of germfree piglets, ferrets, or mice (Hu and Mobley, 1990; Eaton et al., 1991; Andrutis et al., 1995).

*Helicobacter pylori* urease (HPU) accounts for ~10% of total cell protein content (Suzuki et al., 2007). HPU is a large protein, consisting of a dodecameric organization of two subunits (HpUreA, 26.5 kDa; HpUreB, 61.7 kDa; Ha et al., 2001). Structure vs. activity relationships of the non-enzymatic properties of ureases have been so far poorly characterized (Carlini and Ligabue-Braun, 2016). It has been reported that HpUreB interacts with CD74 on gastric epithelial cells inducing IL-8 production (Beswick et al., 2006) and it also binds to Th17 lymphocytes (Zhang et al., 2011). A monopartite nuclear localization signal is present in HpUreA (sequence _21_KKRKEK_26_), and the protein is able to target the nuclei of COS-7 (Lee et al., 2012) and of AGS gastric epithelial cells, causing alterations of the cellular morphology (Lee et al., 2015). Additionally, *H. pylori* secreted outer membrane vesicles (OMVs) contain urease-related proteins, including HpUreA and HpUreB (Olofsson et al., 2010). Incubation of AGS gastric epithelial cells with *H. pylori* OMVs promoted the translocation of HpUreA into the cell cytoplasm and nuclear localization of the protein (Olofsson et al., 2010).

Epidemiological studies have shown that *H. pylori* infection correlates positively with several extragastric pathologies, such as intestine bowel diseases, cardiovascular and cerebrovascular diseases (Franceschi et al., 2015; Goni and Franceschi, 2016; Kyburz and Muller, 2017). Several hematological diseases such as primary immune thrombocytopenia, iron deficiency anemia, childhood leukemia, and coagulation disorders have been associated with *H. pylori* infection (Papagiannakis et al., 2013). The role of this pathogen (Christodoulou et al., 2011) and of its virulence factors in these extragastric diseases is still controversial, requiring further studies (Muhammad et al., 2017).

We have previously reported that canatoxin (Carlini and Guimaraes, 1981), an isoform of *Canavalia ensiformis* urease (Follmer et al., 2001), presents biological properties that are independent of its enzyme activity, including neurotoxicity, activation of blood platelets (Carlini and Guimaraes, 1981; Carlini et al., 1985; Ghazaleh et al., 1997) and *in vivo* pro-inflammatory activity (Benjamin et al., 1992; Carlini and Ligabue-Braun, 2016; Olivera-Severo et al., 2017). We have also demonstrated that a recombinant HPU activated platelets through a lipoxygenase-mediated pathway, leading to exocytosis of dense granules and release of adenosine diphosphate (ADP), which then promoted platelet aggregation (Wassermann et al., 2010). Independently of its enzyme activity, HPU displays a potent lipoxygenase-dependent chemotactic effect on neutrophils, both *in vivo* and *in vitro*, causing cell migration in levels comparable to those induced by fMLP (Uberti et al., 2013). HPU activated human neutrophils eliciting extracellular ROS production and protected neutrophils as well as cultured gastric epithelial cells against apoptosis, interfering on the levels of mitochondrial proteins regulating this process (Uberti et al., 2013; Olivera-Severo et al., 2017). Recently we reported on the angiogenic potential of HPU, a property that could have implication in the invasion and metastization of gastric tumors (Olivera-Severo et al., 2017).

Platelets are anucleated cells involved, aside of hemostasis and thrombi formation, in physiological processes such as tissue regeneration, angiogenesis, inflammation and immunity (Jurk and Kehrel, 2005; Farndale, 2009; Vieira-de-Abreu et al., 2012). Platelets store distinct compounds in granules that, once released, contribute to the immune response (Vieira-de-Abreu et al., 2012) and they produce IL-1β, a pro-inflammatory cytokine (Lindemann et al., 2001; Morrell et al., 2014). IL-1β is considered a platelet agonist, which acts through an autocrine loop to link thrombosis and immunity, signaling to endothelial cells and promoting neutrophil adhesion (Brown et al., 2013). Platelet activation leads to a sustained synthesis of the pro-IL-1β protein, which accumulates in the platelets' cytosol until cleavage to release the cytokine (Lindemann et al., 2001). Platelets interact with components of the subendothelial matrix, with different immune cells and many pathogens (Clemetson, 2011; Morrell et al., 2014) and participate in cancer metastasis (Jurk and Kehrel, 2005; Farndale, 2009). Platelets signal the innate immune system through the release of microparticles and NET formation (Italiano et al., 2010; Carestia et al., 2016). These various characteristics of platelets enable a large spectrum of possible cell interactions and modulation by different membrane receptors (Cimmino and Golino, 2013; Thomas and Storey, 2015).

Collagen, the main component of the subendothelial matrix, promotes adhesion and activation of platelets. Several platelet collagen receptors are involved in these events (Clemetson and Clemetson, 2001). Among them, glycoprotein VI (GPVI), a member of the immunoglobulin superfamily of receptors (Clemetson et al., 1999; Jandrot-Perrus et al., 2000), plays a crucial role in the responses of platelets to collagen. GPVI participates in platelet adhesion to the subendothelium matrix and triggers a collagen-induced activation, resulting in a thromboxane A_2_- and ADP-mediated aggregation thus providing a procoagulant surface for thrombin formation (Moroi et al., 1989; Nieswandt et al., 2001; Nieswandt and Watson, 2003). Collagen binding to GPVI receptor involves signaling through the 12-lipoxygenase pathway (Coffey et al., 2004a,b). On the other hand, when platelets are exposed to low collagen doses, activation through GPVI leads to a secretory phenotype accompanied by release of their dense- and alpha granules' contents, but without changing the platelets into their prothrombotic state (Ollivier et al., 2014).

In this study, we aimed to elucidate the mechanism underlying the activation of platelets exposed to an enzymatically active HPU holoenzyme and to its subunits HpUreA and HpUreB. To that aim, we performed aggregation assays and investigated the roles of platelet's glycoprotein VI and IIbIIIa in HPU-induced and subunits-induced responses. Furthermore, we analyzed expression of P-selectin and the processing of pre-mRNAs in platelets treated with these proteins.

## Materials and methods

### *Helicobacter pylori* urease (HPU)

A recombinant *Helicobacter pylori* urease (HPU) was produced by heterologous expression in *Escherichia coli* BL21 (DE3)-RIL transformed with a PGEM-T-easy (Promega) plasmid carrying the whole urease operon (kindly provided by Dr. Barbara Zambelli, Universitá di Bologna, Italy). HPU was purified from bacterial extracts according to Olivera-Severo et al. (2017). Protein homogeneity was checked by 0.1% sodium dodecyl sulfate 10% polyacrylamide gel electrophoresis (SDS-PAGE) (Figure S1A). Previous to the experiments, a 0.5 mg protein.mL^−1^ solution was dialyzed against 20 mM sodium phosphate 150 mM sodium chloride, pH 7.5 (PBS 7.5), and the buffer from the last dialysis change was used as a negative control in the bioassays.

Fluorescent HPU was prepared by incubation of a 1.0 mg.mL^−1^ solution of urease with 0.1% fluorescein isothiocyanate (FITC) in PBS 7.5 for 60 min at 4°C. The mixture was exhaustively dialyzed against PBS 7.5 and then applied into a Fast-Desalting column (Amersham Biosciences) to remove any unbound FITC (Wassermann et al., 2010).

### *Helicobacter pylori* urease isolated subunits

The subunits of *Helicobacter pylori* urease, HpUreA and HpUreB, were produced in *Escherichia coli* BL21 (DE3)-RIL transformed with pET101/D-TOPO (Thermo Fisher Scientific) plasmids containing the cDNA encoding each of the subunits (a generous gift from Dr Cesare Montecucco, Universitá di Padova, Italy). Bacteria were grown in Luria broth with the antibiotics chloramphenicol (Sigma Aldrich, USA; 40 μg.mL^−1^) and ampicillin (Sigma Aldrich, USA; 100 μg.mL^−1^) for maintenance of the plasmids. The His-tagged subunits HpUreA and HpUreB were purified from bacterial extracts as follows: after cultivation, cells were harvested by centrifugation, suspended in extraction buffer (20 mM sodium phosphate, 500 mM sodium chloride, 5 mM imidazole, pH 7) and lysed using an ultrasonic homogenizer (10 pulses of 30 s) in an ice bath. After centrifugation (20 min, 20,000 × g, 4°C), the homogenates were submitted to Ni^2+^ affinity chromatography on a 5 mL Chelating Sepharose Fast Flow column (GE Healthcare Life Sciences). After removal of the unbound proteins, the column was eluted stepwise with 300 mM imidazole in 20 mM sodium phosphate, 500 mM sodium chloride, pH 7.0 to obtain HpUreA- and HpUreB-enriched fractions. For the last step of purification, the fractions from the Ni^2+^-affinity step were concentrated in Amicon devices with 10 kDa cut-off (Millipore, Belford, MA, USA), applied into a Superdex S200 Hi-load column mounted in a Akta Purifier system (GE Healthcare Life Sciences), equilibrated in PBS 7.5. The protein fractions were analyzed by SDS-PAGE and by Western blots (Figure S1B), pooled and concentrated in Amicon devices (10 kDa cut-off) to a concentration of 0.5 mg.mL^−1^.

### Protein determination

The protein content of samples was determined by absorbance at 280 nm, or by the Coomassie dye binding method (Bradford, 1976).

### Urease activity

The urea hydrolyzing activity of HPU was measured by the alkaline nitroprussiate method (Weatherburn, 1967) using ammonium sulfate as reference, to follow the purification of the holoenzyme.

### Platelet aggregation

Rabbit platelets were used in the aggregation assays. Animal care and handling followed international guidelines and all protocols were approved by the institutional Ethics Committee (UFRGS process 721.217). Platelet aggregation assays were performed essentially as described by Wassermann et al. (2010) using two different turbidimetric assays. For platelet aggregation assays performed in a plate reader (SpectraMax® M3, Molecular Devices), agonists were added to 100 μL aliquots of PRP, without or with inhibitors, to make 150 μL final reaction volume in 96 wells flat bottom plates, with absorbance readings at 650 nm every 11 s during 20 min. For platelet aggregation using a Lummi-aggregometer (Chrono-Log Corp.), after pre-incubation of PRP (300 μL) for 2 min under continuous stirring at 37°C, aggregation was triggered by addition of the agonist (maximal volume 30 μL) and the reaction was registered during 10 min. In both assays, platelet's response was quantified as area under the aggregation tracings. Response to the physiological agonist collagen (bovine tendon, 50 μg.mL^−1^) or ADP (10 μM) (final concentrations) was taken as positive control of platelet activation.

In some of the experiments, platelets were pre-incubated at room temperature without stirring with HPU subunits, inhibitors or antibodies before addition of the platelet agonist: eicosanoid synthesis inhibitors esculetin (0.5 and 1 mM) and indomethacin (75–300 μM); antibodies against platelet glycoproteins GPVI or IIbIIIa, or anti-platelet cell markers (see the following sections for more details).

### Platelet isolation

Peripheral human blood of healthy volunteers was obtained in the presence of 0.313% (w/v) sodium citrate. Written informed consent was obtained from the participants of this study. All procedures regarding blood collection and handling were conducted in strict accordance to Brazilian legislation (Law no. 6.638/1979) and were approved by the institutional Ethics Committee (UFRGS process 721.217).

Human platelets were isolated according to Ollivier et al. (2014) with some modifications; briefly, a platelet-rich plasma (PRP) was separated from whole blood by centrifugation at 200 g for 15 min. For flow cytometry assays (see below), the platelets were pelleted from PRP by an additional centrifugation step (800 × g, 10 min), and washed 3 times (800 × g, 10 min) with a modified Tyrode's buffer (3.6 mM citric acid, 0.5 mM glucose, 0.5 mM KCl, 0.1 mM MgCl_2_, 10.3 mM NaCl, 2 mM CaCl_2_ pH 6.5). After the third wash, the platelets were suspended in the reaction buffer (5 mM HEPES, 12 mM NaHCO_3_, 137 mM NaCl, 2 mM KCl, 2 mM CaCl_2_, 0.3 mM NaH_2_PO_3_, 1 mM MgCl_2_, 5.5 mM glucose, pH 7.4). For quantitative PCR, washed platelets were immunolabeled as CD61 positive cells and separated in a MidiMACS LS column (Miltenyi Biotec) according to manufacturer's instructions.

### RNA extraction and cDNA synthesis

For RNA extraction, the isolated platelets were suspended in reaction buffer to a final cell concentration of 10^7^.mL^−1^. Platelets were incubated with 50 μg.mL^−1^ collagen or 100 nM urease, HpUreA or HpUreB, in reaction buffer, without stirring (to avoid aggregation), at 37°C, for 30, 90, and 180 min. Total RNA was extracted immediately after the stimuli, using TriZol reagent (Ludwig Biotec), following the manufacturer's instructions. cDNA synthesis from total RNA was performed with the High-Capacity cDNA Reverse Transcription kit (Applied Biosystens/ThermoFischer Scientific), following the manufacturer's instructions, using random primers.

### Pro-inflammatory expression profile analysis

Real time quantitative PCR was performed to compare CD14, interleukin-1 beta (IL-1β), cyclooxygenase-2 (COX-2), intercellular adhesion molecule 1 (ICAM-1), and inducible nitric oxide synthase (iNOS) genes expression levels in platelets challenged by collagen, the holoenzyme HPU, and by the HpUreA and HpUreB subunits, in three different time intervals at 37°C, without stirring. The beta actin gene was used (Zsori et al., 2013) to normalize the RNA content of the samples. Gene specific primers were designed to span exon junctions (Table S1). Reactions were carried in an Eco thermocycler (Illumina), using the qPCR-Sybr Green kit (Ludwig Biotec), and following the parameters: 95°C for 5 min (initial denaturation), 40 cycles at 95°C for 10 s (denaturation), 60°C for 15 s (annealing), 72°C for 15 s (extension). Melting curves were performed at the end of each reaction, with temperatures ranging from 55 to 95°C (increments of 0.1°C/s). All cDNA samples were diluted 1:5. Reactions were carried out in technical quadruplicates from two independent biological replicates. Results were analyzed by the 2^ΔΔC_T_^ method (Livak and Schmittgen, 2001).

### Flow cytometry analysis

Platelets (5 μL of PRP) kept at 37°C without stirring were stimulated with 100 nM or 300 nM HPU, 25 μg.mL^−1^ collagen, for 1, 5, and 10 min. Resting platelets (negative controls, no addition) and stimulated platelets in 50 μL of reaction buffer were stained with FITC-labeled anti-CD42 (GP1b) (1:25) (Abcam) and PerCP-labeled anti-CD62P (P-selectin) (1:5) (Abcam) antibodies for 20 min, at room temperature in the dark. After the incubation period, platelets were fixed with 1% paraformaldehyde. Platelets were identified by gating on platelets' size on the basis of forward scatter (FSC) and side scatter (SSC), followed by CD42 expression, a platelet marker. A total of 20,000 events were analyzed for MFI/percentage of CD62P expression. Data were acquired using a FACSCanto II flow cytometer (Becton Dickinson) with BD FACSDiva software and analyzed by Flowjo® vX.

In another set of experiments, platelets were previously treated with polyclonal anti-GPVI (1:10) (Santa Cruz Biotech) or monoclonal anti-IIbIIIa (1:10) (Santa Cruz Biotech) for 20 min at room temperature and then stimulated with 100 nM FITC-conjugated HPU for 1, 5, and 10 min at 37°C. Platelets not treated with the antibodies nor exposed to HPU served as negative controls. After the incubation period, cells were fixed with 1% paraformaldehyde. Platelets were identified as described before. A total of 50,000 events were analyzed for percentage of FITC-positive cells. Data were acquired using a FACSCanto II (Beckton Dickinson) with BD FACSDiva software and analyzed by Flowjo® vX.

### Statistical analysis

The statistical significance of the differences between two groups was assessed using the unpaired Student's *t*-test. For multiple comparisons a two-way analysis of variance (ANOVA) was performed, and the Tukey *post hoc* test was used to calculate significance. GraphPad Prism6 software (San Diego, CA, USA) was used to perform statistical analysis. Statistically significance was set at *p*-value ≤ 0.05. Data in graphs represent mean ± standard error of the mean (SEM) of at least three experiments, unless otherwise stated.

## Results

### Effects of HPU subunits on platelet aggregation

**Figure 1:**
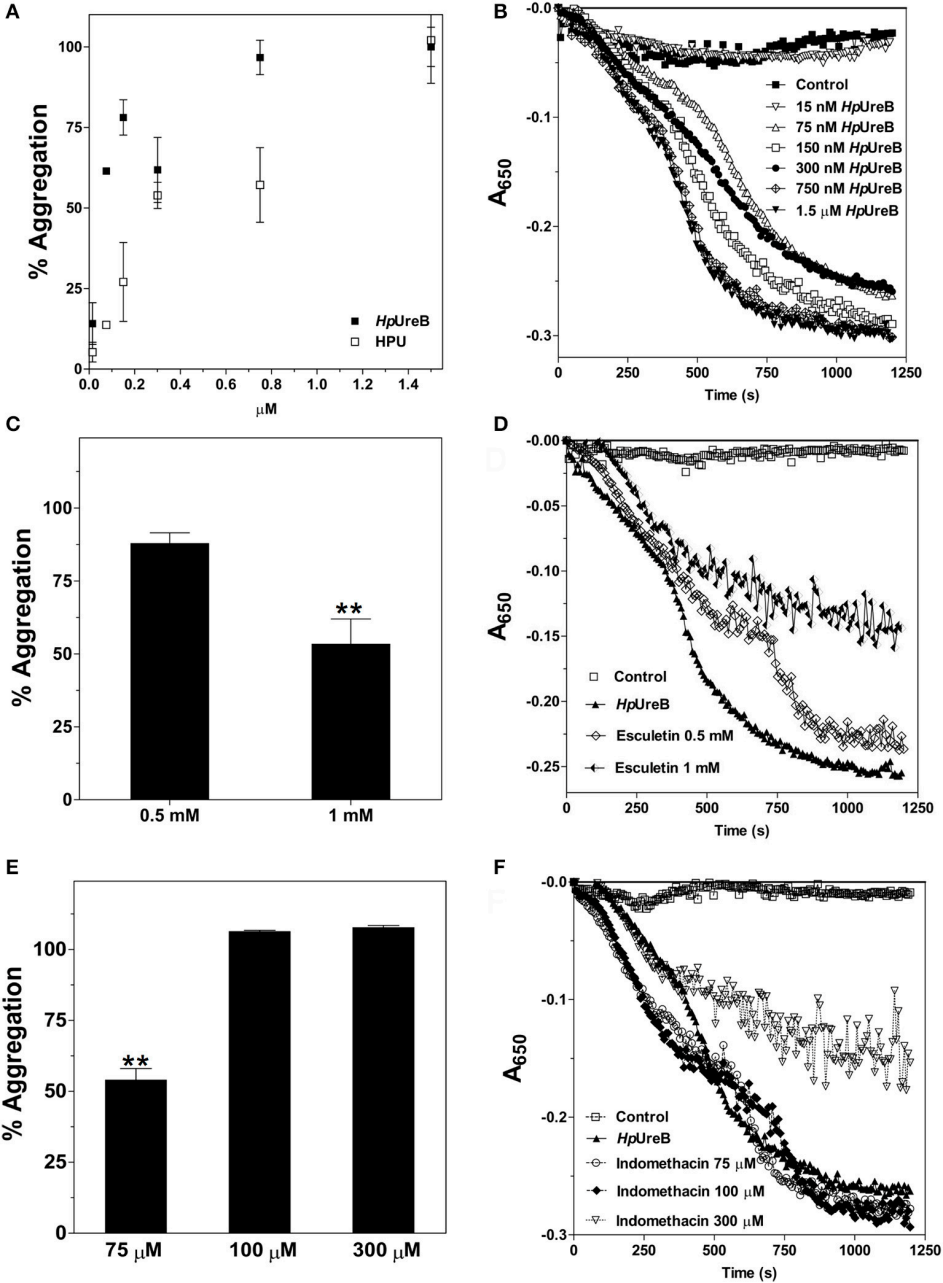
Effects of HPU subunits on platelet aggregation. **(A,B)** HpUreB induces platelet aggregation in a dose dependent manner. **(A)** Aggregation of rabbit platelets was induced with the indicated final concentrations of HPU (open symbols, results taken from Wassermann et al., 2010) and HpUreB (closed symbols, this work). Aggregation induced by HPU or HpUreB at 1.2–1.4 μM was considered 100%. **(B)** Superimposed tracings of aggregation induced by different HpUreB concentrations as measured in the plate reader. **(C–E)** HpUreB-induced platelet aggregation depends on lipoxygenase-derived eicosanoid(s). Platelets were pretreated with the inhibitors of eicosanoids synthesis, esculetin (a 12-lipoxygenase inhibitor) **(C,D)** and indomethacin (a cyclooxygenase inhibitor) **(E,F)** at room temperature for 5 min without stirring. Aggregation was triggered by addition of 750 nM HpUreB (time zero), and after 2 min at 37°C stirring was turned on. Platelets response was monitored on SpectraMax M3 plate reader, with readings at 650 nm every 7 s for 20 min. Superimposed individual tracings of typical experiments are shown in **(B,D,F)**. Aggregation responses were quantified as area under the tracings using SotfMax Pro 5.4.1 **(A,C,E)**. Data are expressed as means ± SEM. Statistical significance was determined by ANOVA followed by Tukey-Kramer test. Values of ^**^p < 0.01.

**Figure 2:**
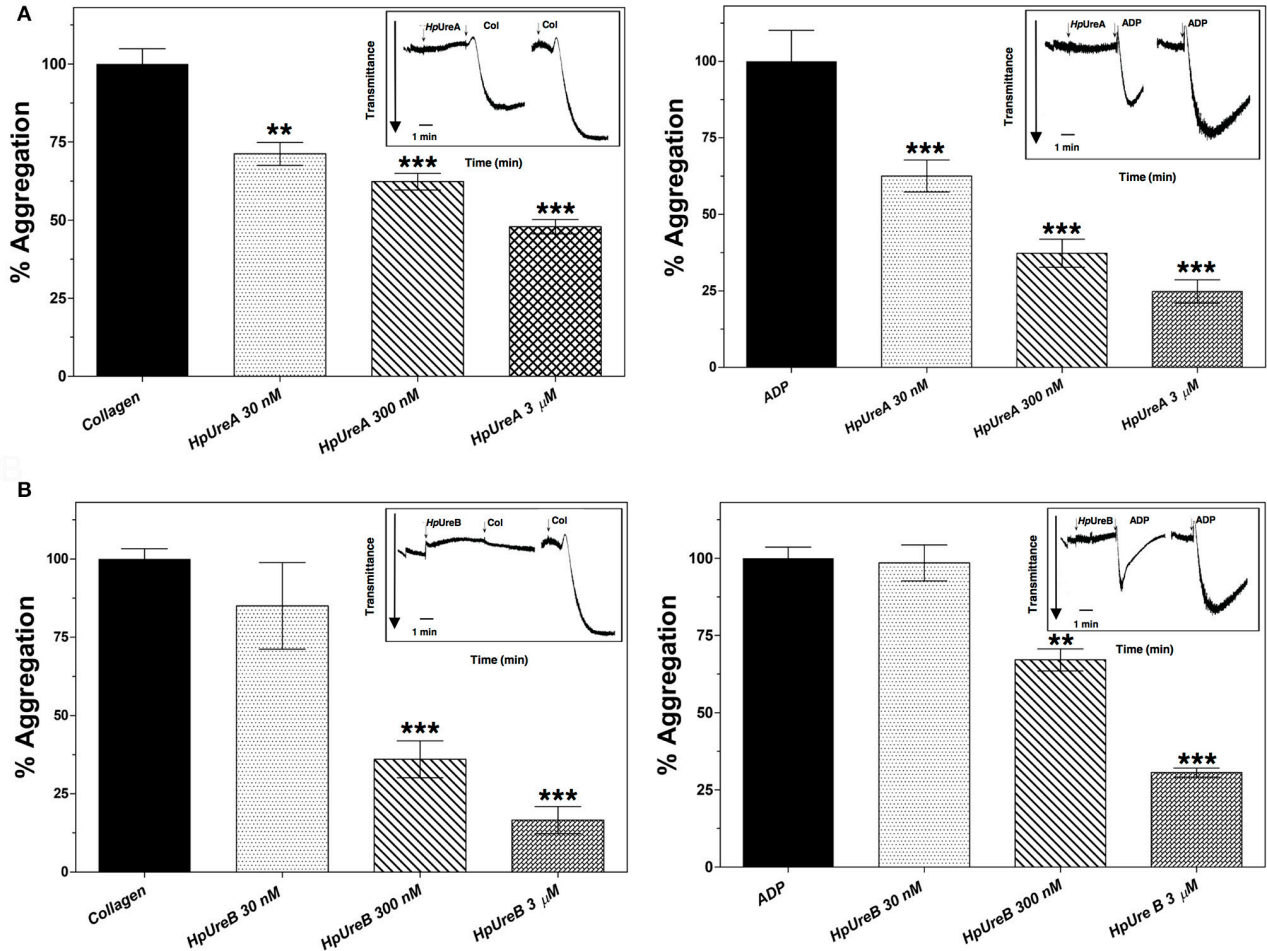
HPU isolated subunits interfere on platelet aggregation triggered by collagen or ADP. Pretreatment with HpUreA **(A)** or HpUreB **(B)** interferes in a dose-dependent manner on the response of rabbit platelets to the physiological agonists collagen (left panels) and ADP (right panels). Platelets were pre-incubated for 5 min at 37°C without stirring with increasing concentrations of HpUreA or HpUreB. Aggregation was then triggered with collagen (50 μg.mL^−1^) or 10 μM ADP. The results are expressed as a percentage of maximum aggregation produced by collagen or ADP. Aggregation was monitored in a plate reader SpectraMax® M3 (Molecular Devices), with readings every 11 s for 20 min at 650 nm. Typical recordings of the aggregation responses are shown in the insets. In all panels, the aggregation responses were quantified as area under the tracings using SoftMax Pro 5.4.1. Data are expressed as means ± SEM. Statistical significance was determined by ANOVA followed by Tukey-Kramer test. Values of ^**^p < 0.01, ^***^p < 0.001.

**Figure 3:**
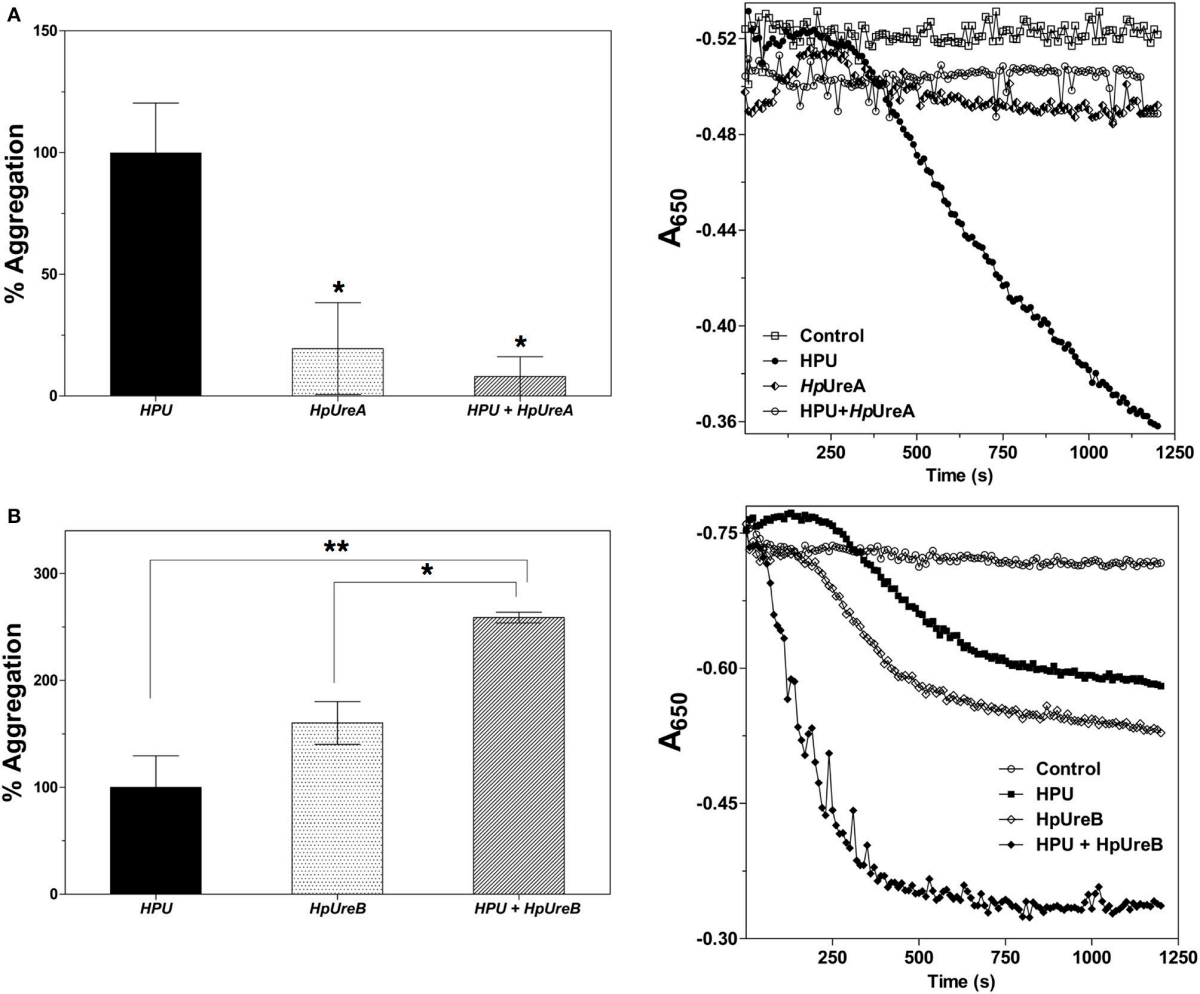
HPU subunits interfere on HPU-induced platelet aggregation. Pretreatment of rabbit platelets with HpUreA **(A)** or HpUreB **(B)** interferes on platelet aggregation induced by 300 nM HPU. Platelets were pre-incubated for 5 min at 37°C without stirring with 1 μM HpUreA or HpUreB and then challenged with 300 nM HPU. Platelets' responses are expressed as percentage of the maximum aggregation produced by HPU (left panels). Aggregation was monitored in a plate reader SpectraMax® M3 (Molecular Devices), with readings every 11 s for 20 min at 650 nm. Superimposed tracings of individual experiments are shown in the right panels. The aggregation responses were quantified as area under the tracings (SoftMax Pro 5.4.1). Data are expressed as means ± SEM. Statistical significance was determined by ANOVA followed by Tukey-Kramer test. Values of ^*^p < 0.05, ^**^p < 0.01.

Our previous study has shown that HPU induces aggregation of rabbit platelets in nanomolar concentrations (Wassermann et al., 2010). To test if the isolated subunits also induce this effect, HpUreA and HpUreB were tested as platelet agonists. While HpUreB induced platelet aggregation in a dose dependent manner (Figures 1A,B), HpUreA had no activity under the same conditions. As reported for HPU, aggregation induced by HpUreB in rabbit platelets also depends on the production of lipoxygenase-derived eicosanoids (Figures 1C,D), as indicated by the inhibitory effect of esculetin, which blocks the platelet 12-lipoxygenase, and by the potentiating effect of the cyclooxygenase inhibitor, indomethacin (Figures 1E,F) (Wassermann et al., 2010). To investigate whether the isolated subunits could interfere in the aggregation triggered by the physiological agonists collagen or ADP, the platelets were previously incubated without stirring with HpUreA or HpUreB, and then challenged with the agonists (Figure 2). Surprisingly, considering the lack of direct effect of HpUreA, both subunits caused a dose-dependent inhibition of platelets' response to the agonists (Figure 2A). HpUreB was more effective than HpUreA in inhibiting platelet's response to collagen or ADP (Figure 2B). Moreover, HpUreB apparently interfered in the second wave of aggregation, abruptly blocking the progression of aggregation in response to released ADP (Figure 2B, right panel), an effect not seen in HpUreA-treated platelets. HpUreA and HpUreB also interfered in the HPU-induced platelet aggregation (Figure 3). While 1 μM HpUreB had a synergistic effect on platelets activated by 300 nM HPU, increasing the aggregation response by 150% (Figure 3A), 1 μM HpUreA decreased the aggregation response by 75% (Figure 3B).

### HPU interaction with platelet membrane receptors

**Figure 4:**
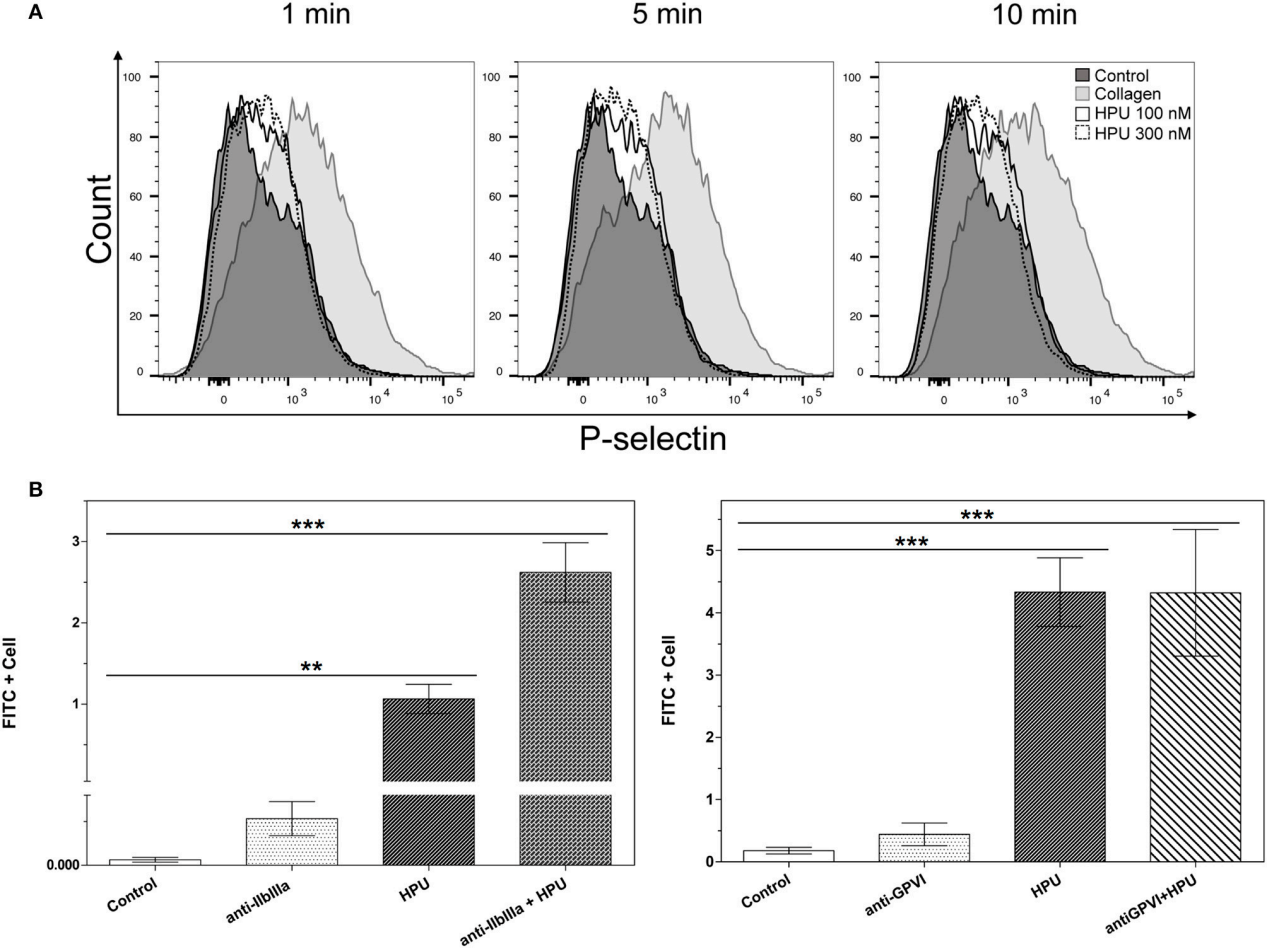
Urease interaction with platelet membrane receptors. Representative flow cytometry histograms of P-selectin exposure on platelets' membranes of non-treated (control), collagen- or HPU-treated cells, at three different times (1, 5, and 10 min), are shown in **(A)**. The bar graph in **(B)** shows the mean percentage of FITC positive cells. Platelets were pre-treated with anti-GPVI or anti-IIbIIIa for 20 min and then challenged with 50 nM FITC-labeled HPU. Data are means ± SEM. Statistical significance was determined by ANOVA followed by Tukey-Kramer test. Values of ^**^p < 0.01, ^***^p < 0.001.

Activation of human platelets by 100 and 300 nM HPU induced P-selectin exposure in a subpopulation of these cells (Figure 4A). This effect occurred immediately after addition of HPU and persisted for at least 10 min. Other signs of activation were also seen, such as increase in the cellular size and membrane complexity (Figure S4). Previously we have hypothesized (see Wassermann et al., 2010) that, in rabbit platelets, HPU may recruit the same signaling pathway triggered by collagen. Considering that anti-GPVI antibodies almost completely blocked HPU-induced aggregation (Figure S2), here we investigated whether HPU interacts with platelets receptors. For that aim pretreatments of human platelets with either anti-GPVI, the main collagen receptor, or anti-GPIIbIIIa, implicated in platelets' activation by fibrinogen and von Willebrand factor, were carried out. Surprisingly, after blockade of GPIIbIIIa by the antibodies, there was an increase in HPU binding to platelets (Figure 4B, left panel). We could not observe reactivity of the employed commercial polyclonal anti-GPIIaIIIb antibodies against HpUreB under our experimental conditions (Figure S3). Nonetheless, the signs of platelet activation (increased cell size) could no longer be seen, thereby confirming that HPU-induced platelet activation somehow involves binding to the platelet membrane, if not directly to, at least in the vicinity of, this physiologically relevant receptor. On the other hand, the binding of FITC-labeled HPU to platelets was not affected by either mono- or polyclonal antibodies against GPVI (Figure 4B, right panel).

### Urease and its subunits modify pre-mRNA processing in platelets

**Figure 5:**
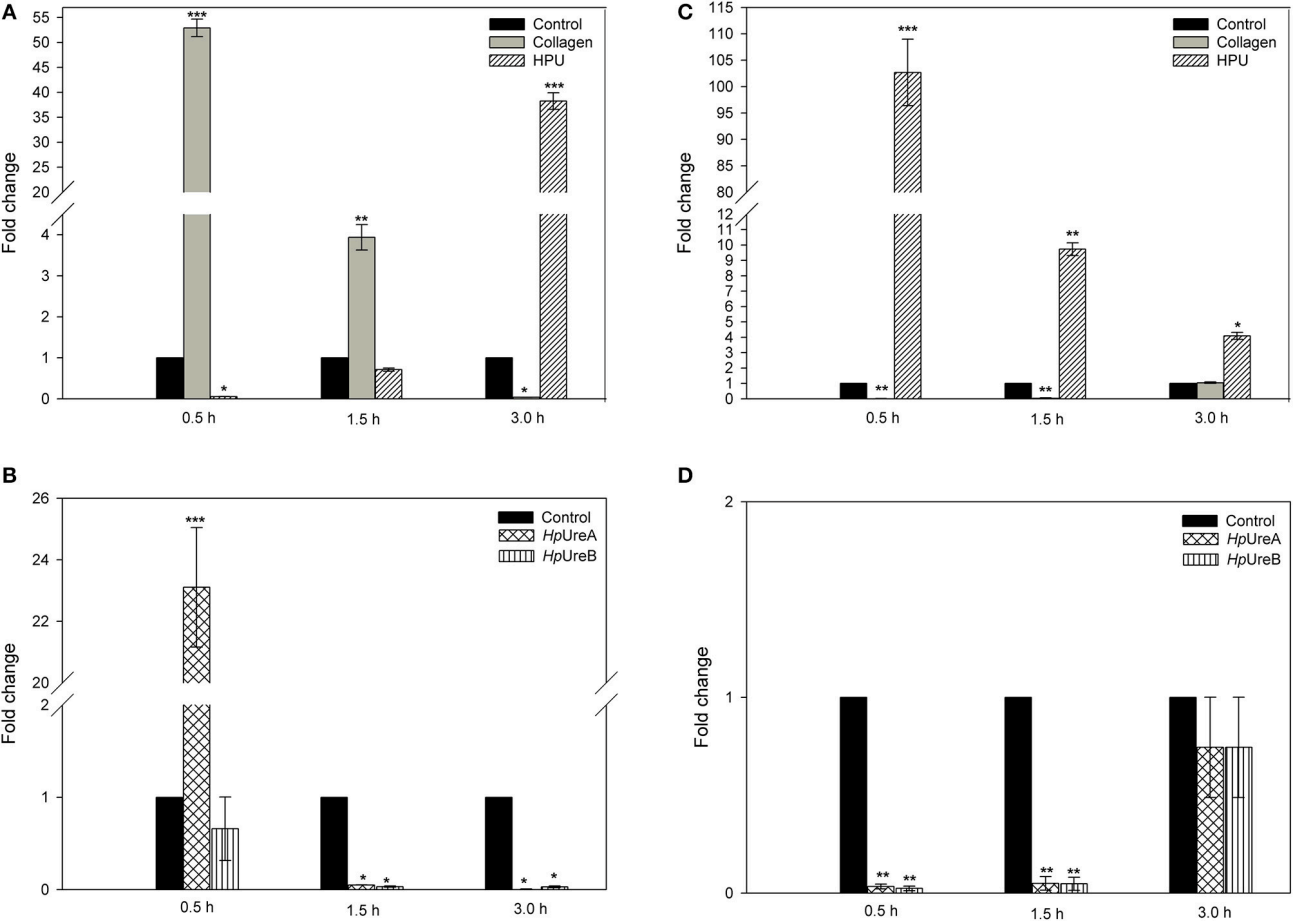
HPU and its isolated subunits modify human platelet's mRNA processing. Platelets were treated with 50 μg.mL^−1^ of collagen, 100 nM HPU, 100 nM HpUreA, or 100 nM HpUreB for 0.5, 1.5, and 3 h at 37°C, without stirring. Untreated platelets were taken as the negative control. mRNA processing was evaluated by RT qPCR using primers designed to span exon junctions. **(A,B)** IL-1β mRNA processing. **(C,D)** CD 14 mRNA processing. Results are shown as means ± SEM of three independent biological replicates, performed in quadruplicates. A *t*-test was performed to compare treatments with the control (buffer). Values of ^*^p < 0.05, ^**^p < 0.01, ^***^p < 0.001.

To evaluate the processing of pre-mRNA in platelets, qPCR analysis was performed after platelets treatment with HPU or its subunits. Collagen-activated platelets were used as positive controls. Collagen increased the IL-1β mRNA processing levels (Figure 5A), peaking at 30 min of treatment and then gradually decreasing to reach levels below the controls in 3 h-treated platelets. Differently, HPU-treated platelets decreased the process in the short treatment (30 min) and then gradually increased IL-1β pre-mRNA processing to reach, after a 3 h treatment, levels similar to those seen in collagen-treated platelets at 30 min (Figure 5A). Exposure of platelets to either HPU's subunits modified the IL-1β mRNA processing, but with different patterns as compared to that produced by the holoenzyme. The time-course of HpUreA's effect was similar to that seen in collagen-exposed platelets. On the other hand, treatment of platelets with HpUreB inhibited IL-1β mRNA processing in all tested times (Figure 5B).

Contrasting with the inhibition observed in collagen-treated platelets (Figure 5C), the processing of CD14 mRNA was greatly enhanced (about 100-fold) in HPU-treated plateletes (30 min). The levels gradually decreased to reach levels still 4-fold higher than in resting platelets with 3 h of HPU treatment. Similar to the inhibition patterns seen in collagen-stimulated platelets, HpUreA or HpUreB inhibited the processing of CD14 mRNA up to 1.5 h after treatment, returning to control levels after 3 h (Figure 5D). Neither HPU nor its subunits interfered on the processing of pre-mRNA of COX-2, ICAM-1, or iNOS under the tested conditions.

## Discussion

The association of *H. pylori* infection, a pathogen with a global dispersal, with several extragastric pathologies has not gone unnoticed. Besides its well established role in stomach diseases, gastric and duodenal cancer (Ferlay et al., 2013), epidemiological studies have associated this bacterial infection with cardiovascular and thromboembolic conditions (Manolakis et al., 2007; Papagiannakis et al., 2013) and other extragastric diseases (Franceschi et al., 2015; Goni and Franceschi, 2016; Kyburz and Muller, 2017). The potential contribution of the very abundant bacterial urease to the pathogenesis of these extragastric diseases in *H. pylori* positive patients has been so far mostly overlooked.

Microbial and plant ureases have many non-enzymatic properties, mostly based on induction of exocytosis and recruitment of eicosanoids pathways, among which are neurotoxicity and pro-inflammatory activity (Carlini and Ligabue-Braun, 2016). One of these non-enzymatic properties of ureases, regardless of their source and quaternary structures, is their ability to induce aggregation of blood platelets (Follmer et al., 2004; Carlini and Ligabue-Braun, 2016). This platelet-aggregating effect of ureases is due to their exocytosis-inducing activity which leads to the release of ADP from platelets' dense granules, and requires the eicosanoid 12-hydroxy-peroxy-eicosatetraenoic acid (12-HPETE), produced by the platelet 12-lipoxygenase (Carlini et al., 1985; Olivera-Severo et al., 2006a,b; Wassermann et al., 2010). These data suggested that platelet aggregation induced by HPU resembles that of collagen-activated platelets through its GPVI receptor, a response reported to also require 12-HPETE synthesis by the activated platelets (Coffey et al., 2004a,b). Back then, we hypothesized that ureases and collagen may recruit similar or overlapping signaling cascades to exert their actions in platelets (Wassermann et al., 2010).

Here, we aimed to deepen the knowledge on how HPU interacts with platelets. Recombinant versions of HPU, and of its two subunits, HpUreA and HpUreB, were produced and platelet aggregation assays, flow cytometry and quantitative PCR were used to analyze how these proteins affect platelets' physiology.

In Wassermann et al. (2010), we reported that 1 μM HPU induced maximal aggregation of rabbit platelets. The response of rabbit platelets to HPU involved the 12-lipoxygenase, as it could be blocked by esculetin, and the eicosanoid 12-HETE, an oxidized derivative of 12-HPETE, was detected in the medium (Wassermann et al., 2010). Here we showed that HpUreB induces rabbit platelet aggregation in the same molar range as that of the holoenzyme. In contrast HpUreA, even in a 10-fold greater concentration had no platelet aggregating activity. This result reinforces the non-enzymatic nature of the platelet-activating property of HPU, since HpUreB has no enzymatic activity (Ha et al., 2001; Suzuki et al., 2007). Platelets' response to HpUreB required production of lipoxygenase-derived eicosanoids, as could be deduced from the dose-dependent inhibitory effect of esculetin (Figures 1C,D). The fact that pretreatment of platelets with the cyclooxygenase inhibitor indomethacin enhanced platelets' reactivity to HpUreB (Figures 1E,F) is expected for a lipoxygenase-mediated process, in a condition of increased availability of arachidonic acid, which is the substrate of both enzymes (Wassermann et al., 2010). These data can be interpreted as HpUreB being the HPU's domain active on platelets, and corroborates previous findings showing that this effect does not require the enzyme's ureolytic activity.

Pre-incubation of platelets with either one of the HPU's subunits inhibited, in a dose-dependent manner, the aggregation response to collagen or ADP (Figure 2). These data indicate that (1) although not inducing platelet aggregation, HpUreA also interacts with platelets; (2) HPU binds to platelets' membranes through at least two binding sites, one present in each of its subunits; (3) the isolated subunits bind to platelets in the same sites as does HPU itself; and (4) the binding of HPU's subunits partially blocks physiological functions of platelets. While an explanation for these observations is not trivial, the data clearly indicated that the binding sites and/or the type of binding to platelet membrane of the two subunits are quite different, implying that distinct mechanisms of action may underly their effects on platelets. The reason why pre-incubation with either HPU's subunits inhibited the platelet aggregation promoted by collagen or ADP is unclear, considering their effects on HPU-induced aggregation (Figure 3). HpUreA inhibited HPU-induced aggregation, which is consistent to its binding to platelets with an antagonist-like behavior. This effect may be related to fact that ureases insert themselves into lipid bilayers thereby affecting membrane physicochemical properties (Piovesan et al., 2014; Micheletto et al., 2016). On the other hand, HpUreB probably interacts specifically with some platelet receptor, as it induces aggregation *per se*, and it acted synergistically with HPU to amplify the aggregating effect (Figure 3).

Activation of human platelets (constitutively positive for CD42, or glycoprotein 1b) by HPU in comparison to collagen was studied by flow cytometry following the expression of P-selectin (CD62P) (Figure 4). Platelets are known to modulate their degranulation and release of specific granules according to the stimulus (Jonnalagadda et al., 2012). For instance, collagen-activated platelets, when in low concentrations of the agonist, can degranulate and release their granular contents without a significant exposure of P-selectin (Ollivier et al., 2014). Here we demonstrated that HPU-stimulated human platelets, despite their low expression of P-selectin, show signs of activation, and eventually aggregate under favorable conditions.

Fibrinogen binding to GPIIbIIIa is a requirement for ADP-induced platelet aggregation. We have previously reported that platelet aggregation induced by canatoxin required ADP release and the presence of fibrinogen (Carlini et al., 1985; Follmer et al., 2001). Here we showed that pre-treatment of platelets with antibodies against GPIIbIIIa enhanced the binding of FITC-labeled HPU to the cells (Figure 4B, left panel). Altogether, these results implicate the involvement of GPIIbIIIa in the ADP-dependent platelet-aggregating effect of ureases. Muhammad et al. (2017) reviewed the mechanisms by which *H. pylori* infection could lead to cardiovascular and thromboembolic diseases, emphasizing that a cross-reactivity between HpUreB and GPIIIa could be the link associating *H. pylori* infection to immune thrombocytopenia. Bai et al. (2009) produced monoclonal antibodies against a recombinant HpUreB and showed that these antibodies cross-reacted with GPIIIa from normal platelets and partially inhibited aggregation induced by ADP. The binding motif recognized by the monoclonal anti-HpUreB in the platelet GPIIIa was not identified. This cross-reactivity of HpUreB and GPIIIa may be implicated in our observation that platelets pre-treated with anti-GP IIbIIIa bound more FITC-labeled HPU than did unstimulated platelets (Figure 4B). The lack of effect of the anti-GPVI antibodies in preventing HPU binding to platelets does not go against our previous hypothesis that HPU (Figure 4B, right panel), and now including HpUreB, share with collagen a lipoxygenase-mediated pathway of platelet activation (Wassermann et al., 2010). However, binding to GPVI seems not to be the feature that explains the similarity of platelets' response to these agonists.

It is well known that platelets participate in the inflammatory process by modulating the activity of other inflammatory cells (Thomas and Storey, 2015; Koenen, 2016). We have previously demonstrated that HPU displays pro-inflammatory activity in the mouse paw edema model, causing an intense eicosanoid-dependent neutrophil infiltration in the tissues (Uberti et al., 2013). In the same study, HPU showed a non-enzymatic, lipoxygenase-dependent, chemotactic effect on human neutrophils, and induced extracellular production of reactive oxygen species (ROS) by the activated cells (Uberti et al., 2013). Our data reinforced results obtained by other groups showing that purified HPU elicited the production of ROS and inflammatory cytokines by human macrophages and primary monocytes *in vitro* (Harris and Granger, 1996; Shimoyama et al., 2003), induced transendothelial migration of T cells (Enarsson et al., 2005) and increased the expression of inducible NO synthase (Gobert et al., 2002). All these activities of HPU contribute to tissue inflammation and injury. Here we investigated whether activation of human platelets by HPU leads to a pro-inflammatory phenotype of these cells.

Platelets have reservoirs of pre-mRNA that are processed into the corresponding mRNA upon stimulation (Denis et al., 2005). Treatment of platelets with collagen, HPU, HpUreA, or HpUreB, modified their processing of IL-1β and CD14 pre-mRNAs, each protein producing effects with a distinct kinetics. Collagen and HpUreA had similar time pattern for stimulation of IL-1β pre-mRNA processing, with peaks at 30 min after treatment and then decreasing to levels below control after 3 h (Figures 5A,B). The slower but persistent effect of HPU of increasing IL-1β production 3 h after treatment confirms that this protein changes platelets into a pro-inflammatory phenotype. Further studies are necessary to confirm if the pre-mRNA processing changes observed here impact as well the platelets' protein levels of IL-1β and CD-14 after exposure to HPU.

Zhang et al. (2009) reported that platelets express TLR4, CD14 and MyD88, the signaling pathway triggered in response to lipopolysaccharides (LPS), as described in other cell types (Funda et al., 2001). CD14 is a co-receptor of various Toll-like receptors (TRLs) present in hematopoietic and non-hematopoietic cells that recognizes PAMPs (pathogen-associated molecular patters), triggering the innate immune response and participating in inflammation (Zanoni and Granucci, 2013). Distinct from collagen and the subunits HpUreA and HpUreB, that lowered CD14 pre-mRNA processing below control levels, HPU greatly enhanced the processing of CD14 mRNA (Figure 5). This result suggests that HPU activates at least part of the signaling pathway triggered by LPS, and the increased expression of CD14 in HPU-activated platelets corroborates their phenotype conversion into pro-inflammatory cells. The absence of CD14 expression in unstimulated platelets, or in platelets stimulated by collagen under normal physiological conditions, in contrast to the increased levels after HPU stimulus, resembles the reaction of intestinal mucosal cells, which only show increased expression levels of CD14 associated to inflammatory processes (Funda et al., 2001).

Similar to cell activation through LPS where a role of CD14 in the induction of TNF-α, IL-1β, IL-6, and IL-8 expression has been identified (Dentener et al., 1993; Schumann et al., 1994), here we hypothesize that CD14 expression may be modulating the IL-1β levels in platelets stimulated with HPU. This does not occur when platelets are stimulated with collagen, HpUreA or HpUreB. It has been described that platelets stimulated by LPS (Brown and McIntyre, 2011) or dengue virus (Hottz et al., 2013) process IL-1β pre-mRNA and that once stimulated, these cells release microparticles loaded with IL-1β by a mechanism not yet completely elucidated (Hottz et al., 2013). If HPU-stimulated platelets also release microparticles containing IL-1β, that could be delivered nearby endothelial cells or immune cells responsive to IL-1β, such as macrophages and lymphocytes, will be a subject of future studies.

The natural existence of any urease's subunits in a free state has never been described thus the relevance of the biological effects described here for HpUreA or HpUreB is uncertain. On the other hand this study provided valuable insights into the structure vs. activity relationships of HPU concerning its effects on platelets. While it became clear that HpUreB carries the major “platelet-active” domain of HPU, the contributions of HpUreA to the platelet activating effect of HPU are less clear. The antagonistic effect of HpUreA against aggregation induced by the physiological agonists ADP and collagen, proved its interaction with platelets' membranes. Moreover only HpUreA increased IL-1β pre-mRNA processing like HPU did. Thus both HPU subunits contribute to the protein's effect on platelets although in different ways. Another evidence of the existence of two platelet binding sites in HPU is the fact that both of its subunits competed with HPU, partially blocking the aggregating effect of the holoenzyme. In alignment with this conclusion, other studies by our group performed with the isolated subunits of the tri-chained *Proteus mirabilis* urease have identified a “platelet-activating” domain in one of its small subunits whose sequence is homologous to the C-terminal half of HpUreA (Broll V, unpublished results).

In summary, in this work we have shown that HPU and its subunits affect platelet physiology in ways that may contribute to the pathogenicity of *H. pylori* by other mechanisms besides enabling bacterial survival in the gastric lumen. The pro-inflammatory phenotype of HPU-activated platelets implies altered participation of these cells in many physiological processes, possibly contributing to the development of the extragastric diseases associated to *H. pylori* infection. Altogether our results reinforce the importance of microbial ureases, acting also in non-enzymatic ways, as a virulence factor of pathogenic microorganisms.

## Author contributions

AS-G and DO-S planned and conducted experiments on the interactions of urease and its subunits with platelets; AS-G, DO-S, and AFU planned and conducted platelet aggregation assays; AS-G, DO-S, AFU, and NC-S produced the recombinant proteins; AS-G and FS planned and conducted mRNA processing experiments; NJ and BP conducted flow cytometry assays; AS-G, DO-S, FS, NJ, and CC have written and revised the manuscript; CC has conceived and coordinated this study.

### Conflict of interest statement

The authors declare that the research was conducted in the absence of any commercial or financial relationships that could be construed as a potential conflict of interest.
