# Supplementary figures and images for: The Impact of Helicobacter pylori Urease upon Platelets and Consequent Contributions to Inflammation
Source: Front Microbiol. 2017 Dec 12;8:2447. doi: 10.3389/fmicb.2017.02447 (PMC5733092; doi:10.3389/fmicb.2017.02447)

A

HPU

61 kDa  
→

29 kDa  
→

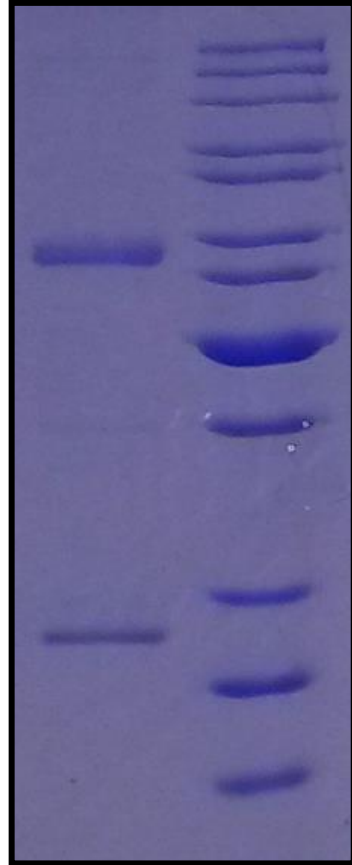

B

HpUreA

29 kDa  
→

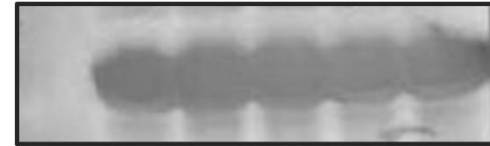

HpUreB

61 kDa  
→

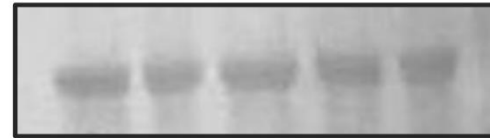

Supplement: Supplementary file 1 [file Image1.PDF]

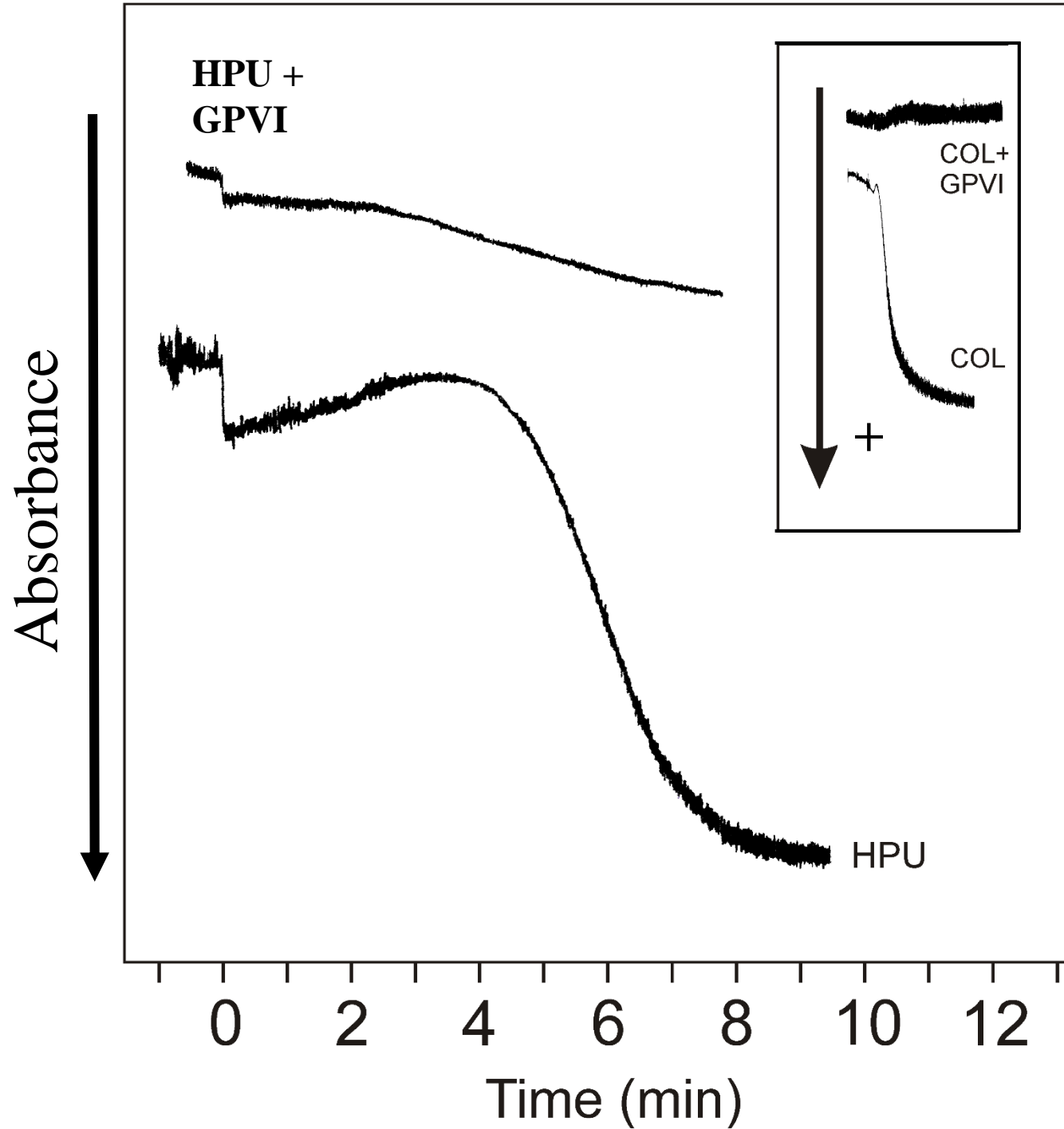

Supplement: Supplementary file 2 [file Image2.PDF]

Anti-GPVI

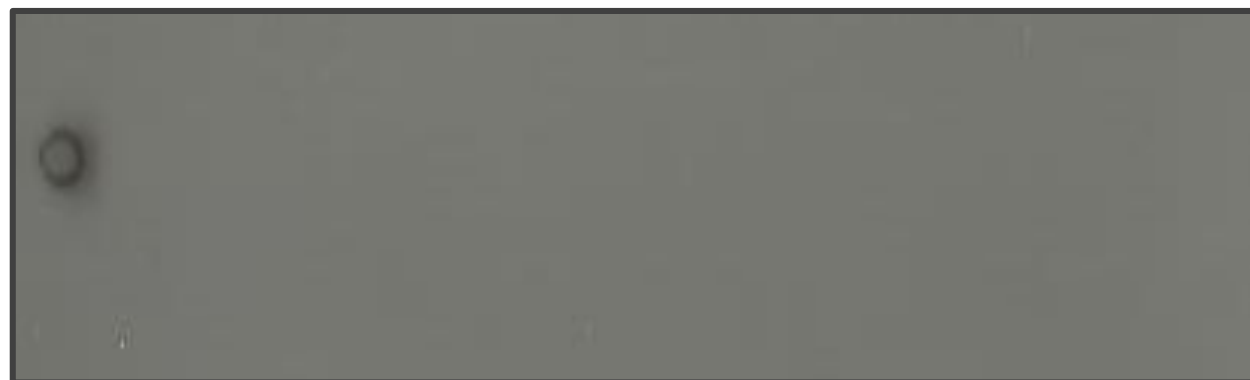

Anti-IIbIIIa

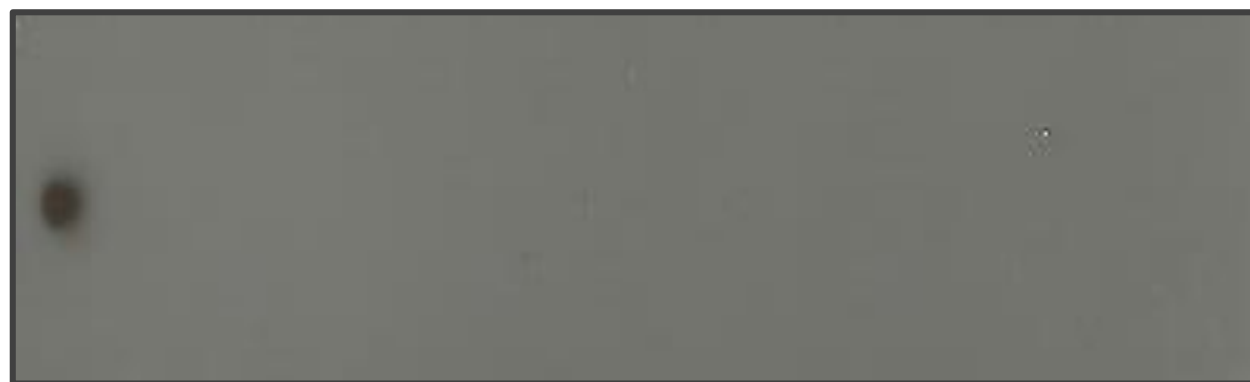

C+

5  $\mu$ g

0.2  $\mu$ g

HPU

Supplement: Supplementary file 3 [file Image3.PDF]

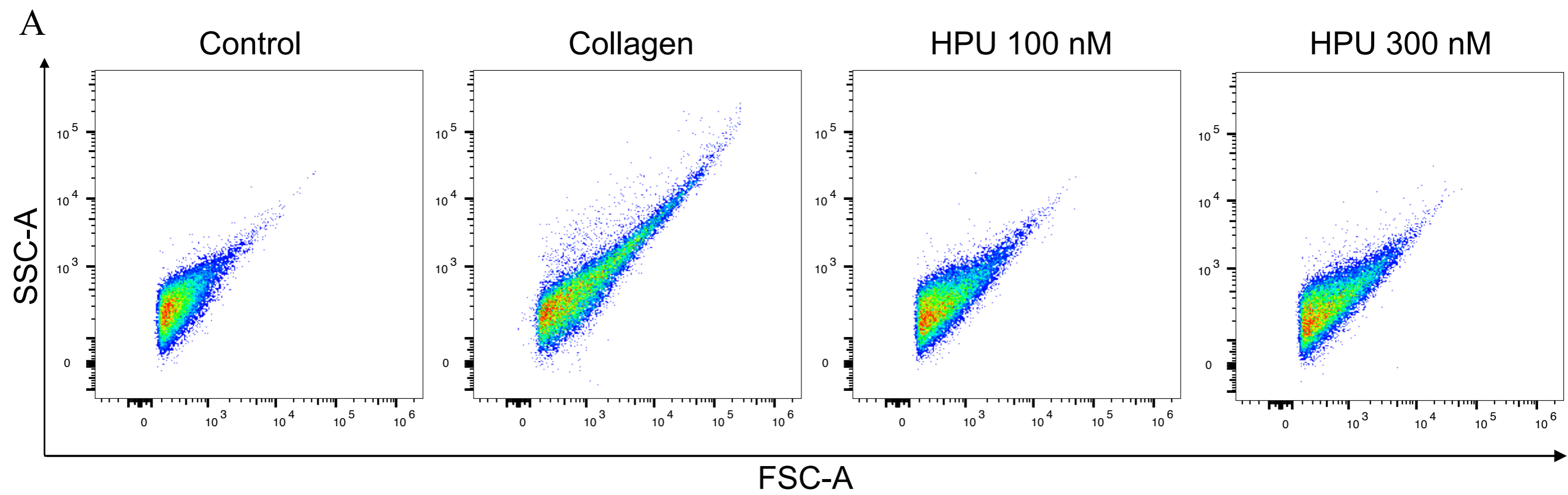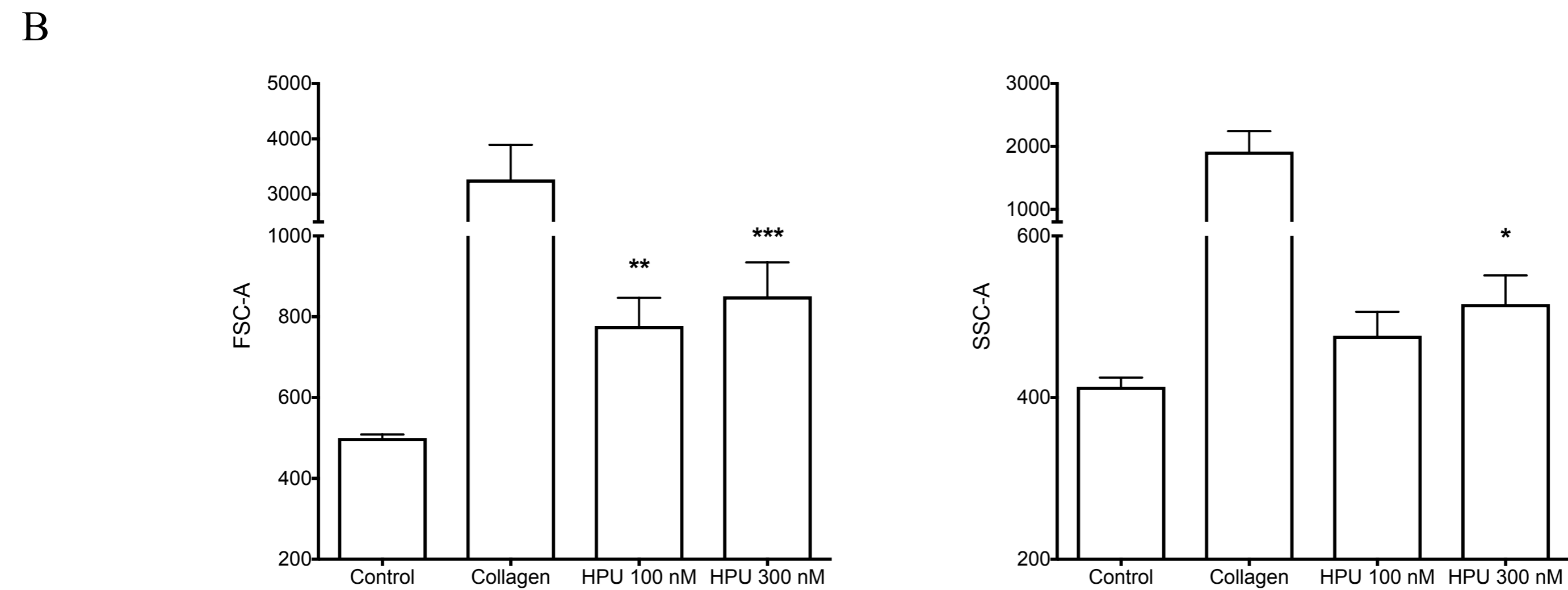

Supplement: Supplementary file 4 [file Image4.PDF]
