# Supplementary material for: The Impact of Helicobacter pylori Urease upon Platelets and Consequent Contributions to Inflammation
Source: Front Microbiol. 2017 Dec 12;8:2447. doi: 10.3389/fmicb.2017.02447 (PMC5733092; doi:10.3389/fmicb.2017.02447)
Supplement: Supplementary file 5 [file Table1.DOC]

**Table S1.**

Primers used in the real time PCR experiments.

| Gene | Primer | Sequence (5’ – 3’) |
| --- | --- | --- |
| IL-1β | Forward | CGTATATGCTCAGGTGTCAACCTATCTTCG |
|  | Reverse | CTTGTTGCTCCACTTGTTGCTCCA |
|  |  |  |
| CD14 | Forward | TATCGACCATGGAGCGCG |
|  | Reverse | CCTCTACTGCAGACACACACT |

| ICAM | | Forward | | CCTCACCGTGTACTTTTATTTTGAGATGGA | |
| --- | --- | --- | --- | --- | --- |
|  |  | Reverse | | TCAGGGGATTCTCCTGGGAG | |
|  | |  | |  | |
| iNOS | | Forward | | GCAGAATGTGACCATCATGG | |
|  |  | Reverse | | ACAACCTTGGGGTTGAAGGC | |
|  | |  | |  | |
| COX-2 | | Forward | | TGAAACCCACTCCAAACACA | |
|  |  | Reverse | | GAGAAGGCTTCCCAGCTTTT | |
|  | |  | |  | |
| Beta actin | | Forward | | GATGGCCACGGCTGCTTC | |
|  |  | Reverse | | TGCCTCAGGGCAGCGGAA | |
|  |  | |  | |  |
