# Supplementary material for: The Impact of Helicobacter pylori Urease upon Platelets and Consequent Contributions to Inflammation
Source: Front Microbiol. 2017 Dec 12;8:2447. doi: 10.3389/fmicb.2017.02447 (PMC5733092; doi:10.3389/fmicb.2017.02447)
Supplement: Supplementary file 6 [file DataSheet1.DOCX]

Supplementary Material

Urease of *Helicobacter pylori* and its interaction with platelets: contributions to the inflammatory process.

Adriele Scopel-Guerra^†^, Deiber Olivera-Severo^†^, Fernanda Staniscuaski, Augusto F. Uberti, Natália Callai-Silva, Natália Jaeger, Bárbara N. Porto, Celia R. Carlini^*^

***Correspondence:** Celia R. Carlini [celia.carlini@pucrs.br](mailto:celia.carlini@pucrs.br)

**Materials and Methods**

**SDS-PAGE ,Western Blot and Dot blot analysis**

The recombinant proteins (30 μg total protein) after the final purification step were denatured in sample buffer (50 mM Tris–HCl, pH 6.8, 1% SDS, 5% 2-mercaptoethanol, 10% glycerol, 0.001% bromophenol blue) and heated in a boiling water bath for 3 min. Samples were resolved in 10% or 12% SDS–PAGE. Rainbow markers (Amersham Pharmacia Biotech) run in parallel were used to estimate molecular masses. Proteins bands in the gels were stained with Coomassie blue. For Western blots, proteins bands in the gels were transferred to PVDF membranes (Hybond-P, Amersham Pharmacia Biotech, the membranes were then blocked with Tween-TBS (20 mM Tris–HCl, pH 7.5, 500 mM NaCl, 0.1% Tween-20) containing 1% bovine serum albumin. Immunoreactivity of the resolved proteins were probed for 2 h with polyclonal rabbit antibodies: anti-HpUreA (Santa Cruz Biotechnology, 1:1000), anti-HpUreB (Santa Cruz Biotechnology, 1:1000), anti-GPVI (Santa Cruz Biotechnology, 1:500) or anti-IIbIIIa (Santa Cruz Biotechnology, 1:100). After exhaustive washing with Tween-TBS, PVDF membranes were incubated with biotin-conjugated anti-rabbit IgG antibodies (1:1000; Santa Cruz Biotechnology) for 1 h followed by incubation with streptavidin-conjugated horseradish peroxidase (1:1000; Caltag Laboratories, Burlingame, CA). Immunoreactive proteins were visualized by 3,3′-diaminobenzidine (Sigma Aldrich, USA) staining

For dot blot assays, 5 μL of purified HPU five-fold serially diluted solutions (1 mg.mL^-1^) were spotted on PVDF sheets. Afterwards, the membranes were blocked and washed as described above for the Western blot analysis, and then probed with anti-GPVI (Santa Cruz Biotechnology, 1:500) or anti-IIbIIIa (Santa Cruz Biotechnology, 1:100) antibodies. Secondary antibodies (anti-rabbit, 1:20.000) coupled to horseradish peroxidase were from Jackson ImmunoResearch Laboratories, Inc. (West Grove, PA). The protein bands were visualized using a chemiluminescence detection kit (Millipore, MA, USA).

**Figure S1. Purification of HPU, HpUreA and HpUreB.** (A) SDS-PAGE of purified recombinant HPU showing two protein bands, corresponding to HpUreA (29 kDa) and HpUreB (61 kDa). (B) Western blots of HpUreA (29 kDa) and HpUreB (61 kDa) bands probed with the corresponding antibodies. Each lane corresponds to the purified proteins isolated from different bacterial colony purification fractions.

**Figure S2. HPU-induced platelet aggregation is inhibited by anti-GPVI.** Platelet aggregation induced by HPU or collagen (inset) is blocked in the presence of anti-GPVI antibodies. Platelets were pre-incubated with anti-GPVI (500 μg.mL^-1^) for 10 min at room temperature without stirring, and then aggregation was triggered (time zero) by addition of HPU (300 nM) or collagen (20 μg.mL^-1^). Aggregation was monitored continuously by absorbance readings during 8 min. A typical result is shown out of at least 4 experiments using platelets from different rabbits.

**Figure S3. Anti-IIbIIIa and Anti-GPVI do not cross-react with HPU.** Dot blot analysis to show the absence of immunoreactivity of the anti-GPVI and anti-IIbIIIa antibodies towards HPU. Decreasing quantities of HPU (1, 0.20 and 0.04 mg.mL^-1^) were adsorbed (5 μL) onto the PVDF membranes. C+ indicates addition of the antibodies directly to the PVDF sheets. Dot blots were revealed using a chemiluminescence detection kit and the images were digitalized.

**Figure S4. HPU induces platelet activation.** (A) Representative flow cytometry dot plots of platelets population of non-treated (control), collagen- or HPU-treated cells. The bar graph in (B) shows variations of cell size and membrane complexity. Platelets were treated with 25 μg.mL^-1^ collagen, 100 nM or 300 nM HPU. Data are means ± SEM. Statistical significance was determined by ANOVA followed by Tukey-Kramer test. Values of p < 0.01*, <0.001 **.
